# Reliable measurement of *E. coli* single cell fluorescence distribution using a standard microscope set-up

**DOI:** 10.1186/s13036-017-0050-y

**Published:** 2017-02-20

**Authors:** Marilisa Cortesi, Lucia Bandiera, Alice Pasini, Alessandro Bevilacqua, Alessandro Gherardi, Simone Furini, Emanuele Giordano

**Affiliations:** 1 Department of Electrical, Laboratory of Cellular and Molecular Engineering “S.Cavalcanti”, Electronic and Information Engineering “G.Marconi” (DEI), University of Bologna, Cesena, Italy; 2 Advanced Research Center on Electronic Systems for Information and Communication Technologies “E. De Castro” (ARCES), University of Bologna, Bologna, Italy; 3 Department of Computer Science and Engineering (DISI), University of Bologna, Bologna, Italy; 4 Department of Medical Biotechnologies, University of Siena, Siena, Italy; 5 BioEngLab, Health Science and Technology, Interdepartmental Center for Industrial Research (HST-CIRI), University of Bologna, Ozzano Emilia, Italy; 6 Present address: SynthSys – Center for Synthetic and Systems Biology and School of Biological Sciences, University of Edinburgh, Edinburgh, UK; 7 Present address: Division of Respiratory Medicine, School of Medicine, University of Nottingham, Nottingham, UK

**Keywords:** Single-cell fluorescence, Fluorescence microscopy, Synthetic biology, Phenotypic noise

## Abstract

**Background:**

Quantifying gene expression at single cell level is fundamental for the complete characterization of synthetic gene circuits, due to the significant impact of noise and inter-cellular variability on the system’s functionality. Commercial set-ups that allow the acquisition of fluorescent signal at single cell level (flow cytometers or quantitative microscopes) are expensive apparatuses that are hardly affordable by small laboratories.

**Methods:**

A protocol that makes a standard optical microscope able to acquire quantitative, single cell, fluorescent data from a bacterial population transformed with synthetic gene circuitry is presented. Single cell fluorescence values, acquired with a microscope set-up and processed with custom-made software, are compared with results that were obtained with a flow cytometer in a bacterial population transformed with the same gene circuitry.

**Results:**

The high correlation between data from the two experimental set-ups, with a correlation coefficient computed over the tested dynamic range > 0.99, proves that a standard optical microscope– when coupled with appropriate software for image processing– might be used for quantitative single-cell fluorescence measurements. The calibration of the set-up, together with its validation, is described.

**Conclusions:**

The experimental protocol described in this paper makes quantitative measurement of single cell fluorescence accessible to laboratories equipped with standard optical microscope set-ups. Our method allows for an affordable measurement/quantification of intercellular variability, whose better understanding of this phenomenon will improve our comprehension of cellular behaviors and the design of synthetic gene circuits. All the required software is freely available to the synthetic biology community (MUSIQ Microscope flUorescence SIngle cell Quantification).

**Electronic supplementary material:**

The online version of this article (doi:10.1186/s13036-017-0050-y) contains supplementary material, which is available to authorized users.

## Background

The rigorous quantification of gene expression is fundamental for the characterization of synthetic gene circuits’ functionality in transformant cells. In this regard the importance of phenotypic variability within an isogenic population has recently emerged [1]. Exploring this facet of synthetic biology requires the ability to monitor mRNA or protein concentrations at the single cell level. These measurements are not achievable with the presently widely used multiplate fluorometers, that generate population level datasets [2] of fluorescent signals from mRNA or protein reporters [3, 4] accounting for an average readout of synthetic gene circuits’ functionality in transformant cells.

Quantification of fluorescent signals at the single cell level is typically achieved using a cytofluorimeter [5]. However, a cytofluorimeter is an expensive apparatus – due to the coupling of optics, fluidics and control software – which makes it hardly affordable in small laboratories. On the other hand, a standard fluorescent microscope set-up is a much more common laboratory equipment, due its reasonable cost in front of its broad general-purpose usefulness. Moreover, when coupled with a microfluidic device, a microscope set-up could be easily adapted to measure the dynamics of fluorescence signals.

**Fig. 1:**
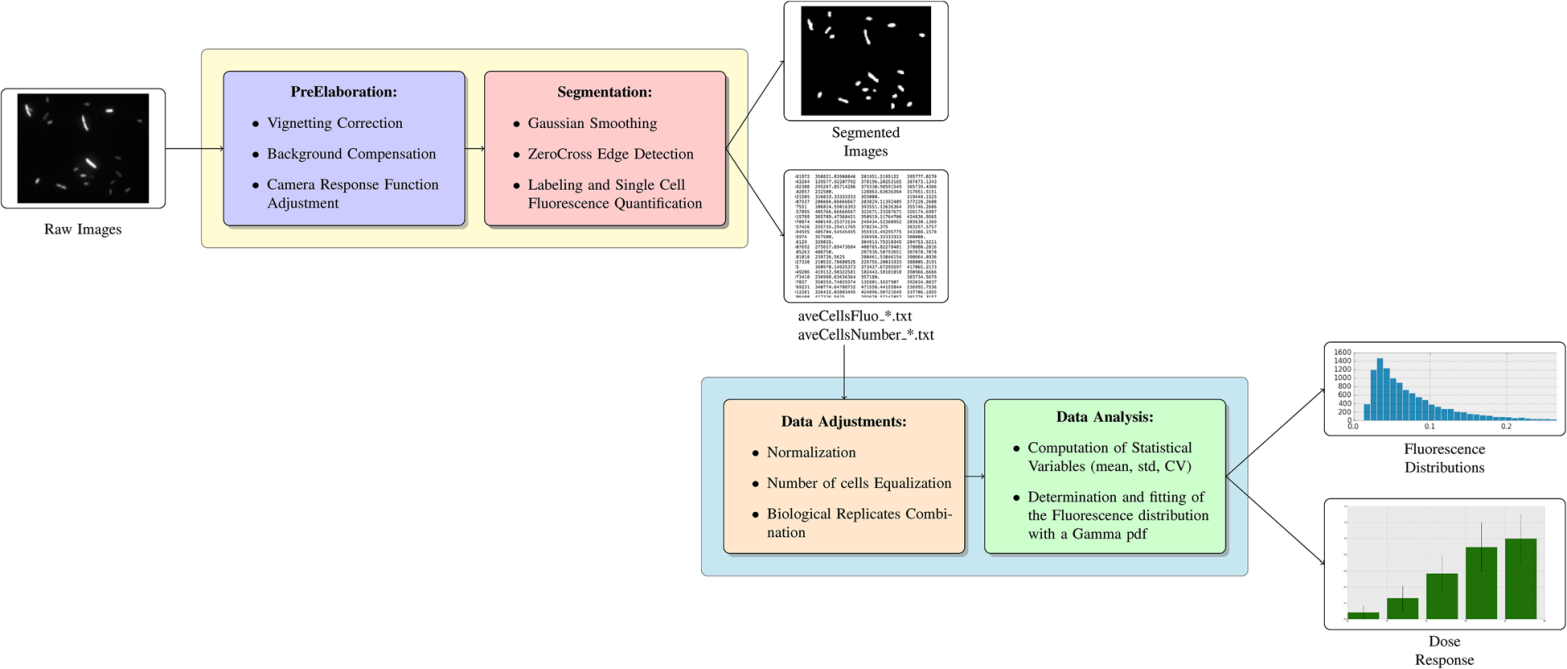
Flowchart representing the image analysis pipeline implemented in our algorithm. The first section of the software (yellow box) is responsible for segmenting the images and extracting the fluorescence intensity emitted by each cell. Beside the segmentation routine, it includes the pre-elaboration phase and its input are the raw images (in 8-bit RGB format). The outputs of this section are i) a pdf file, containing the images at different stages of elaboration, ii) a text file containing each image cell density, computed as number of segmented cells divided by the volume used to prepare the slide and iii) another text file, in which the fluorescence intensities emitted by each segmented cell are reported. This last text file is the input of the second section of the algorithm that is responsible for the analysis of the fluorescence data and for the production of the outputs of our software. It mainly consists in the computation of the statistical modes of the fluorescence distribution (average, standard deviation, CV) and their graphical representation. However it also includes a data analysis step in which the fluorescence intensities are normalized and the cardinality of the populations is equalized

We previously explored [6, 7] how to define an appropriate hardware/software fluorescence microscopy set-up in the perspective of driving the qualitative nature of fluorescent microscopy towards a quantitative accurate measurement of fluorescent signals emitted by populations of bacterial *E. coli* cells transformed with synthetic gene circuits. Here we thoroughly characterize this set-up comparing the output with the data acquired with a flow cytometer in [5]. Our results show a substantial equivalence of both techniques, at least within the tested conditions. Set-up specification, calibration/validation steps description (Fig. 1), and a custom-made software script for image post-processing is presented and offered to the free availability (http://www.mcbeng.it/en/downloads/software/musiq.html) of users of synthetic biologsts.

## Methods

### Sample preparation

**Fig. 2:**
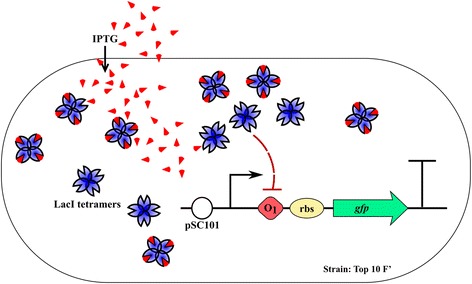
Schematic representation of the synthetic gene circuit used in this work. The expression of the green fluorescent protein (GFP) can be modulated transcriptionally, through the addition of IPTG, due to the presence of the Lac Operator O_1_. The circuit was cloned in a pSC101 low copy number plasmid and transformed in E. coli cells of the strain TOP 10 F’ overexpressing the Lac repressor

The data presented in this paper were obtained testing synthetic gene circuits composed of standard biological parts in the BioBrick format. Gene circuits conformed to the Standard Assembly 10, and were transformed in the TOP10F’ *E. coli* strain. Cells were cultured in M9 medium complemented with the antibiotic ampicillin and glucose as a major carbon source. The circuit used to quantify single cell fluorescence level, previously characterized in [5], includes a reporter fluorescent protein coding sequence (GFPmut3b green fluorescent protein [8]; GFP) cloned in a pSC101 low copy number plasmid. By including an operator site for the lactose repressor the synthesis of GFP can be transcriptionally induced via exogenous Isopropyl β-D-1-thiogalactopyranoside (IPTG) (Fig. 2). Before measuring the fluorescence levels each culture was diluted to an OD_600_ = 0.05 and grown under orbital shaking for 3 h, to reach the mid-exponential phase of growth, at 37 °C in 5 ml of M9 medium in the presence of the appropriate concentration of IPTG. Cell fluorescence signal was measured with: a) a fluorescence microscope and b) a cytofluorimeter. Three biological replicates were considered for each tested condition for both instruments. To compensate the bias introduced by the time lag between the testing of the first and last sample, the acquisition order was varied among the biological replicates. When using the microscopy set-up, the sequences were determined so that the sum of the rankings of each sample over the biological replicates was equal. To further limit the deviation from the desired condition, after the beginning of the acquisition the cultures were stored at 4 °C.

### Microscopy and image processing

#### Microscopy set-up

An inverted Eclipse TE2000-U (Nikon) microscope equipped with a DS-Qi1Mc (Nikon) (Table 1) monochrome digital cooled camera was used to collect brightfield or fluorescent images of culture samples through an S-Fluor 40x 0.9 NA oil/water (Nikon) objective. The proprietary Nis Elements Documentation v 4.20 software (Nikon) was used for image acquisition. A Nikon 78589 - HBO 100 W/L2 mercury lamp was used to generate the excitation light.

**Table 1:** Technical specifications of the DS-Qi1Mc camera that is part of the Microscopy set-up

Image Pickup Device	2/3-inch square pixel, 1.5 megapixel interline CCD
Color/Monochrome	Monochrome
Number of Recording Pixels *2	1280 x 1024
Quantization	12 bits
Sensitivity	Equivalent to ISO 800

#### Image acquisition

To prepare the sample for image acquisition, 500 μL from each cell liquid culture sample were spun down and resuspendend in 100 μL of sterile PBS to reduce the background autofluorescence and to maximize the cardinality of the sampled population while preserving an optimal field of view coverage. A volume of 3 μL of this cell suspension was dispensed over a glass slide and sealed with a coverslip. A minimum of 70 images out of 6 distinct slides were acquired for each sample. During image acquisition, the shutter speed is heuristically defined to distinguish clearly the cells while avoiding loss of information due to saturation. When cells with different average fluorescence are imaged, this parameter needs to be adapted in order to correctly capture both the minimal and the maximal fluorescence intensity values, which may hamper the comparison among samples acquired with different exposure times. Having characterized the camera response function, however, permits to express the fluorescence values in terms of normalized irradiance rather than pixels intensities. This allows to reliably compare samples acquired with a different shutter speed simply dividing the normalized irradiance by the exposure time set during the acquisitions, thus restoring the right relationship between different samples.

### Flow cytometry set-up

A PAS II (Partec) flow cytometer equipped with an argon ion laser was used for bacterial population analysis in [5], using a 488 nm blue line for excitation. Fluorescence emission was acquired in FL1 through a 515–545 nm band pass filter.

## Results

The aim of this work was to define a reliable protocol to measure single cell fluorescence values using a microscope set-up. In this perspective, preliminary experiments were carried out for the appropriate set-up calibration and procedure validation. Data collected using this original protocol were finally compared to the single cell fluorescence level measured with a flow cytometer. The results show a substantial equivalence of both the techniques.

### Calibration Experiments

The signal acquired with a fluorescence microscope is affected by multiple distortions, such as i) vignetting, ii) photobleaching, and iii) non-linearities in signal digitalization. All these artifacts, together with other iv) minor distortions, require appropriate considerations and corrections that are described in the following subsections.

#### Vignetting

Vignetting consists in a reduced brightness at the image edges caused by imperfections in the lenses system. This aberration can be compensated by the pixel-wise addition of each image to a vignetting image acquired with the same set-up. A vignetting image is created acquiring an image of a uniformly emitting field and inverting any recorded intensity variation. In our experimental set-up the vignetting image, obtained using a fluorescent slide, was uniform down to exposure times < 3 ms, where the variability in the pixels’ intensities was likely attributable to shot noise and dark current. Since the shutter speeds used during the experiments were at least ten times longer than this value, we did not apply any vignetting correction in the image analysis pipeline. However, the MUSIQ software includes the routines to compensate this aberration in case a different set-up needs it.

#### Photobleaching

Measuring fluorescence intensities requires the dimming of the signal over time, due to the photochemical destruction of the fluorophore by the excitation light, to be compensated. While with very short exposure times, as in a flow citometry assay, this effect is negligible, bacterial cells over a microscope slide are exposed to longer excitation times that might compromise the detection of the actual fluorescence intensity. This aberration is generally modeled with a negative exponential function whose time constant is estimated through a time-lapse experiment, as shown in [9]. However, the application of this method to the set-up employed in our analysis was unable to isolate the photobleaching effect from a number of confounding factors, such as intracellular pH, exposure time and local environment. Thus we decided to rather set a maximum number of images to be acquired from a single slide within a suitable time limit under the continuous exposure to the excitation light. This granted a negligible fluorescence’s decay. This value was heuristically determined (see segmentation section below) and set to 15 images/slide and 2 min of exposure time. Within these limits, it was possible to detect a sufficient events count without the experimental cost of using an excessive number of slides or compromising the signal’s intensity. The same approach is recommended for the photobleaching control in other experimental set-ups.

#### Non-linearity in signal digitalization

**Fig. 3:**
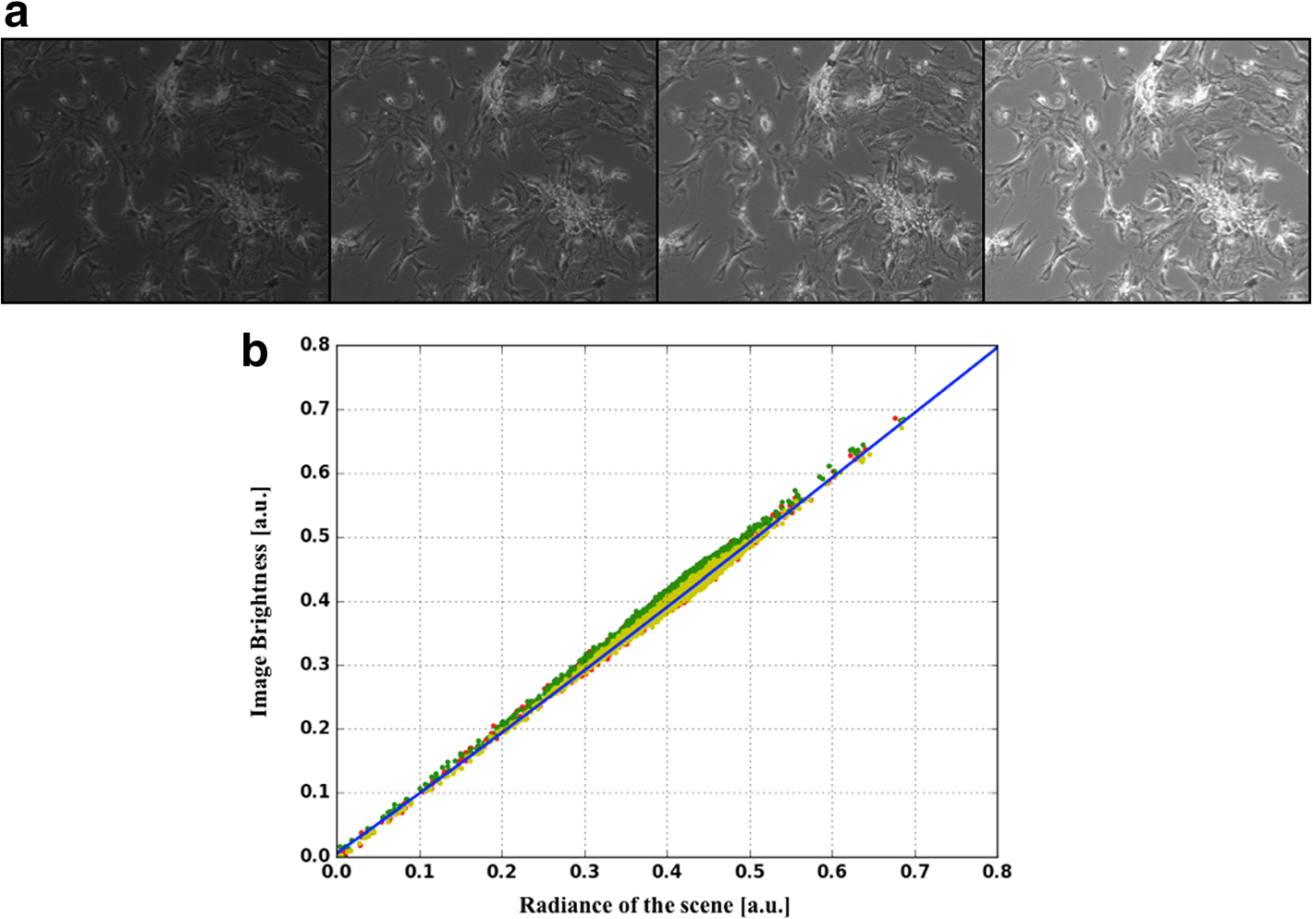
Camera Response Function calibration experiment. In **a** is reported one of the sets of brightfield images of fixed eukaryotic cells used during this preliminary test. The same field of view was captured at different exposure times (between 1 and 4 ms). The third degree polynomial function representing the CRF (blue line) is plotted together with the data on which it was fitted (red, green and yellow dots in **b**). The different colors identify the pixels belonging to each image pair

The camera response function (CRF) describes the transformation of the scene radiance in the raw images’ gray levels. Non-linearities in this analytical relation introduce a distortion in the acquired biological sample, thereby leading to an erroneous reconstruction of its empirical distribution. The standard calibration technique for the CRF is the radiometric self-calibration method [10, 11] that consists in fitting with a polynomial function the variation of intensity assessed in multiple images of the same field, acquired at different exposure times. Since the radiance of the scene is constant, this relation describes how the luminous signal is modified by the camera as a function of the exposure time. Since the CRF depends only on the camera and not on the recorded signal, this approach was applied to our set-up using brightfield images of fixed eukaryotic cells acquired at four exposure times ranging from 1 to 4 ms. The analysis of these images led to the identification of a third degree polynomial that was inverted with the Cardano’s method. An automatic procedure for compensating the distortion of the signal introduced by the camera was defined accordingly (Fig 3). During the parameters identification process, the Tikhonov regularization method, as implemented in the Matlab toolbox regtools [12], was used with a threshold automatically selected using the L-plot. This approach is part of the MUSIQ calibration script.

#### Minor distortions

Images acquired with a fluorescence microscope are affected by other minor distortions (e.g. progressive dimming of the arc lamp, temperature fluctuations). As these factors are very difficult to isolate and characterize, and might unpredictably interact, the use of an internal calibrator sample is part of the MUSIQ protocol. Therefore, a sample is adopted as a reference throughout all the experiments. Since the calibrator undergoes the same distortions of any other target sample, it provides an implicit correction factor. In addition, the use of a calibrator allows the meaningful comparison of experiments performed in different days, data acquired with alternative instruments, or samples with divergent fluorescence intensities. Maximally induced samples were used as a calibrator in our experiments.

### Image processing

#### Pre-processing

The pre-processing step is fundamental for the correct segmentation of the bacterial cells and the reliable quantification of the fluorescence intensity. As shown in Fig. 1, it consists of two main steps: background correction and CRF compensation.

The former removes from the images the spurious fluorescence components (e. g. auto-fluorescence of the media) and it was implemented as described in [13]. The background intensity was evaluated applying a morphological grayscale opening to the image, with a structuring element of the same size or bigger than the foreground elements (the cells). This estimate was then pixel-wise subtracted from the input image. This strategy is simple and yet very versatile since it does not rely on any specific hypothesis on the background features (e.g. its uniformity).

The second step of the pre-processing phase is the CRF compensation. As previously detailed above, the relationship between the image intensity and the radiance of the scene was approximated with a third degree polynomial. This function was inverted, using the Cardano’s method, and then evaluated at the 256 gray levels of the input images. The corresponding values were saved in a text file that the pre-elaboration function uses as a look-up table to efficiently replace each pixel of the image with the corresponding normalized irradiance level.

#### Segmentation

**Fig. 4:**
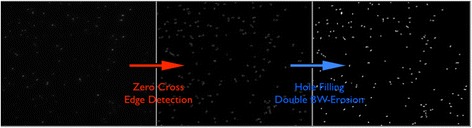
Exemplification of the main steps of the segmentation algorithm. The leftmost image results from the pre-elaboration phase. The application of the zero-crossing edge detection method leads to the central image, in which only the outline of the cells are visible. The rightmost image represents the result of the segmentation procedure, in which all the pixel belonging to a cell, and only those pixels, are different from 0

Bacterial cell segmentation is performed with custom made software whose core is the zero-crossing edge detection method, which is based on the estimate of the null points of the second derivative of the image [14]. This routine is implemented in other image processing libraries coded in different programming languages. We here implement this algorithm into a Python language-based function, to ensure its portability and compatibility with different set-ups. To eliminate the spurious edges and identify the ones that more likely represent the outline of a cell, the zero-crossing algorithm is preceded by a smoothing of the image with a Gaussian filter. Once the boundaries of the bacteria have been identified, the algorithm applies a hole filling procedure and then the segmentation is finalized by a morphological erosion of the resulting image (Figs. 1 and 4), which is applied to equalize the pre- and post-segmented cell size calculated in our set-up. This step might not be required when using other microscopes. Subsequently, the fluorescence intensity of each cell is computed by averaging the value of all the pixels belonging to a certain cell (identified with a labeling routine on the segmented image). Note that in a microscope set-up the density of a bacterial culture can be determined by counting the number of cells in the field of view and relating it to the volume used during the slide preparation (number of cells/μL). This estimate showed a linear relation (*R* ^2^ = 0.996) when compared with the OD_600_ measurement of bacterial cultures at different concentrations, (Additional file 1: Figure S2) and, where of interest, can be used to infer the density of the population from the images employed to evaluate the fluorescence signal.

#### Image and data storage

At the end of the elaboration the output files are saved. A pdf file containing the images at different stages of elaboration and two text files respectively containing i) the fluorescence intensity of each cell and ii) the density of bacterial cells in each image, are saved in the ‘Results’ directory in the same path as the folder containing the images (Fig. 1).

#### Image and data analysis

**Fig. 5:**
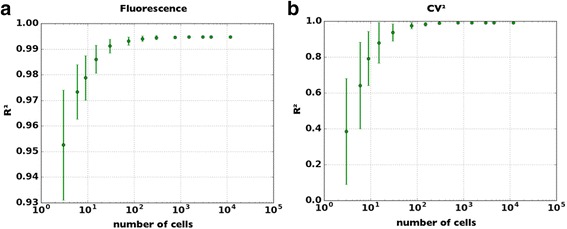
Study of the correlation between the data acquired with the microscope set-up and those evaluated with a flow cytometer. The former dataset was divided in groups of equal cardinality and without common elements, and then the Pearson’s correlation coefficient (R^2^) between each sub-population and the entire flow cytometer dataset was calculated. In **a** the fluorescence data are considered while the CV^2^ data are shown in (**b**)

The protocol described was used for the analysis of all the data presented in this work, including the determination of the values of the heuristic thresholds used to ensure a negligible effect of the photobleaching over data acquired with the microscope set-up. In this regard, the average fluorescence intensities of the first and last third of images acquired from the same slide were compared to evaluate the degradation of the fluorescent signal due to the photochemical destruction of the fluorophore by the excitation light. In our settings, the acquisition of 15 images from the same slide, within the time limit of 2 min, is associated with a negligible decay of the signal and allows the acquisition of a meaningful amount of data in a reasonable time (Fig. 5).

### Single-cell fluorescence measurement

The single-cell fluorescence intensities were measured in engineered *E.coli* cells where the signal can be transcriptionally induced via exogenous IPTG (Fig. 2). This choice is particularly convenient since a modification of the inducer concentration produces a change in the statistical moments of the fluorescence distribution, allowing the set-up validation over a wide range of signal’s intensities using a single gene circuit, thus avoiding biases introduced by different topologies or environmental conditions. Data are expressed as average value ± standard error (SE). The squared coefficient of variation (CV^2^) was used to quantify biological noise, since it is a measure of the signal’s dispersion around its average value. To assess the capability of the proposed method to provide a reliable, low-cost alternative to a flow cytometer, we validated the set-up described above comparing its results with those obtained with a flow cytometer, the gold standard for the acquisition of single-cell fluorescence. Both datasets were normalized with respect to the average fluorescence intensity of the tested circuit at the highest level of induction. The same number of cells (~12 x 10^3^) was used for the acquisition of each induced fluorescence level with the microscopy set-up. This facilitates the comparison among different experimental conditions while preventing distortions introduced by the different cardinality of the tested populations. This number of cells was the minimum value required to obtain a stable relation with flow cytometry measurements (Fig 5a).

**Fig. 6:**
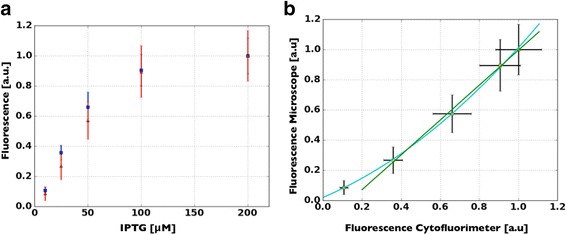
Comparison between the fluorescent signal acquired with the microscope setup and the one obtained with the flow cytometer. In **a** the dose response curve for the tested gene circuit is reported. There is a very good agreement between the results obtained with our set-up (red dots) and those of the flow cytometer (blue dots), with only minor discrepancies at the lower induction levels. In **b** the accordance between the data acquired with the two instruments is analyzed through a correlation graph. The relation is linear (green line, *R* ^2^ > 0.99) and the SEs are comparable, demonstrating the ability of our set-up in capturing, at the single cell level, the average fluorescence intensity emitted by a population of bacteria. A quadratic function fits better the relation between the two datasets at the beginning of the dynamic range, likely due to the higher sensitivity of the flow cytometer (MSE = 7.6x 10^-5^). The equations of the green line and of the parabola that fit the data points are reported below. Fluo_micro_ = 1.15 Fluo_cyto_ -0.16, Fluo_micro_ = 0.44 Fluo_cyto_^2^ + 0.55 Fluo_cyto_ + 0.02

Figure 6a shows a dose response curve as obtained with both the flow cytometer (blue dots) and the microscope (red dots). Similar results are provided by the two approaches, with almost superimposed experimental values. The application of the Mann-Whitney *u* test confirmed that all the induction levels were distinguishable with statistical significance (*p* < 0.01) both with the flow cytometer and the microscope setup. Figure 6b highlights the monotonic second degree relation (cyan line, MSE = 7.6x 10^-5^) between the fluorescence intensities obtained with the two instruments. The relation is almost linear (green line, *R* ^2^ > 0.99) and shows comparable SEs. Only at the lowest induction level this function shows a non-linear behavior, likely caused by the higher sensitivity of the flow cytometer.

**Fig. 7:**
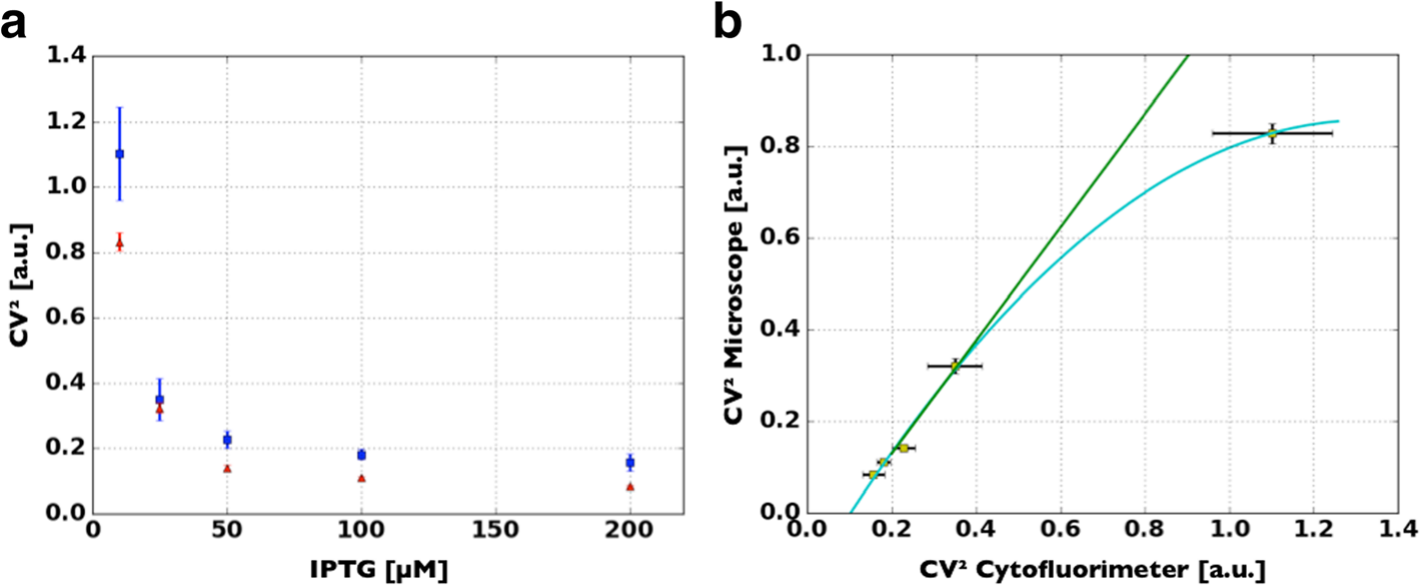
Analysis of the biological variability within an isogenic bacterial population as determined with the flow cytometer and our microscope set-up. In **a** the dependence of the CV^2^ [computed as (σ/μ)2] on the concentration of IPTG is investigated through a dose response curve. The agreement between the data acquired with the alternative set-ups is very good, with only a slight tendency of the microscope setup (red dots) of underestimating the variable of interest, due to the higher dynamic range of the flow cytometer (blue dots). **b** The correlation graph supports these considerations, since the relation between the data acquired with the two instruments has a linear trend (green line *R* ^2^ > 0.99). Only the data point with the highest CV^2^, corresponding to the lowest induction level, deviates from linearity (as presented in Fig. 6b). Again a quadratic function fits better the relation between the two datasets at the rightmost end of the dynamic range, likely due to the higher sensitivity of the flow cytometer (cyan line, MSE = 1.8 x 10^-4^). The linear equation and the parabola that best fit the data points are reported below. CV^2^_Micro_ = 1.28 CV^2^_cyto_ -0.13. CV^2^_Micro_ = -0.61(CV^2^_cyto_)^2^ + 1.58 CV^2^_cyto_ -0.13

The CV^2^ is shown in Fig. 7a, where its dependency upon inducer concentration can be observed. The microscopy set-up (red dots) is able to capture the behavior of this variable of interest, with statistically significant different values associated with distinct induction levels of the synthetic gene circuit tested (Mann Whitney *u* test, *p* < 0.01). These results are coherent with the measurements performed using flow cytometry (blue dots in Fig. 7a), as highlighted by the correlation graph in Fig. 7b. As already remarked for the dose-response curve (Fig. 6b), the second-degree relation between CV^2^ extracted with the two approaches is linear on most of the dynamic range (*R* ^2^ > 0.99) and shows comparable SEs. The lowest induction level, corresponding to the highest CV^2^, is responsible for the nonlinear behavior (MSE = 1.8 x 10^-4^) that is associated to a higher dynamic range of the flow cytometer, which is able to better capture dimmer fluorescent signals.

As observed for the average fluorescence of the cell population, a stable relation between the CV^2^ calculated with the two approaches is observed (Fig. 5b), despite the lower number of cells (~12 x 10^3^) processed with the microscope with respect to the flow cytometer (~5 x 10^4^).

## Discussion

Monitoring synthetic gene circuits’ functionality in transformant cells usually relies upon fluorescent signal-based quantitative analysis of the expression of reporter genes. As population-averaged data might not be representative of the behavior of the tested sample, due to cellular heterogeneity affecting both prokaryotes [14] and eukaryotes [15], the acquisition of the fluorescent signal at the single cell level, and an accurate quantification of its dispersion within the population are required [16].

Here we have presented MUSIQ, a protocol that allows to upgrade, with an appropriate hardware/software configuration, a standard fluorescence microscopy set-up in the perspective of driving the qualitative nature of fluorescence microscopy towards a quantitative accurate measurement of fluorescent signals emitted by single E. coli cells. The complete characterization of our method showed that the results obtained with MUSIQ are remarkably comparable to those of a cytofluorimeter, the most widely used instrument in synthetic biology for the quantification of single cell fluorescence. Even though the throughput of the microscopy set-up was lower, with a difference of about one order of magnitude [5, 17], both the average signal (Fig. 6) and its dispersion (Fig. 7) were correctly quantified by the presented method, with a Pearson’s correlation coefficient above 0.99. By analysing the variation of this parameter with the cardinality of the population, we have demonstrated that few hundreds cells are able to correctly identify fluorescence distribution (Fig. 5), thus improving the usability of our protocol.

The flow cytometer, however, has a higher sensitivity at the lower end of the dynamic range, since the concordance between the two methods decreases when recording dim signals, especially when the variability is considered. The five tested levels of induction, however, were determined to be different with statistical significance and it is important to note that this aspect is also dependent on the technical specifications of the camera used to record the signal.

While being more time consuming and showing an higher dependency on the operator, a fluorescence microscopy set-up allows for a more accurate characterization of the tested synthetic gene circuit, preserving cell morphology and culture’s spatial pattern and enabling the dynamic acquisition of the same cells over time [18, 19] up to the spatio-temporal localization of specific proteins inside individual cells [20]. Furthermore MUSIQ integrates all the procedure required to calibrate a standard fluorescent microscopy set-up, thus giving to our approach the potential to make single cell level quantitative analysis of fluorescent signals accessible to many laboratories, avoiding the need to buy a flow cytometer or a highly sophisticated fluorescence microscopy set-up.

The custom-made software for images post-processing used in this analysis, together with a detailed description of the calibration and validation steps are available @ http://www.mcbeng.it/en/downloads/software/musiq.html.

## Conclusion

The protocol presented in this work can be used to quantify the fluorescent signal at single cell level, with a basic hardware and custom-made freeware software. An optical fluorescence microscope, once thoroughly characterized, exhibits the desired characteristics, with the advantage of being versatile and adaptable to the requirements of the specific experiment. It has significant potential for expansion and customization of protocols and experimental conditions supporting, e.g., the execution of dynamic experiments through the addition to the set-up of a microfluidic device and an incubator chamber. Furthermore, the presented method could be easily adapted to be used in other applications where the output is a fluorescent signal. These include immunofluorescence assays using fluorophore-conjugated antibodies to quantify gene expression in eukaryotic cells, and new diagnostic strategies such as the one presented in [21] where the measurement of the auto-fluorescence in a highly keratinized epithelium can address the analysis of a middle ear pathology.

## Additional file

Additional file 1: MUSIQ user guide. (DOCX 172 kb)
