# Supplementary material for: Reliable measurement of E. coli single cell fluorescence distribution using a standard microscope set-up
Source: J Biol Eng. 2017 Feb 20;11:8. doi: 10.1186/s13036-017-0050-y (PMC5317050; doi:10.1186/s13036-017-0050-y)
Supplement: Additional file 1: — MUSIQ user guide. (DOCX 172 kb) [file 13036_2017_50_MOESM1_ESM.docx]

**MUSIQ User Guide**

In the following, the set of instructions required for extracting and analyzing single cell fluorescence measurements with the MUSIQ software is described. This step-by-step guide will show how to use this tool and adapt it to different optical microscopy set-ups.

Installation and Download:

MUSIQ is released under the Gnu Public Licence (GPL v2) and can be downloaded freely @ www.mcbeng.it/downloads/MUSIQ.

It is mainly coded in Python 2.7.11 and makes use of the following libraries: os, tifffile, scipy.ndimage, numpy, matplotlib.pyplot, matplotlib.backends.backend_pdf.

This part of the software is responsible for image segmentation, fluorescence quantification and data analysis and thus will be executed every time an experiment is made.

However the MUSIQ software includes another part, written in Matlab (R2012a), that is required for the identification of the CRF and thus is required for calibrating the microscopy set-up. This code does not need to be executed before every experiment, as the CRF is considered to be invariant in the setup and thus, once characterized, can be applied to any experiment performed with the same optical microscope.

A schematic representation of the MUSIQ software library is reported in Figure S1.


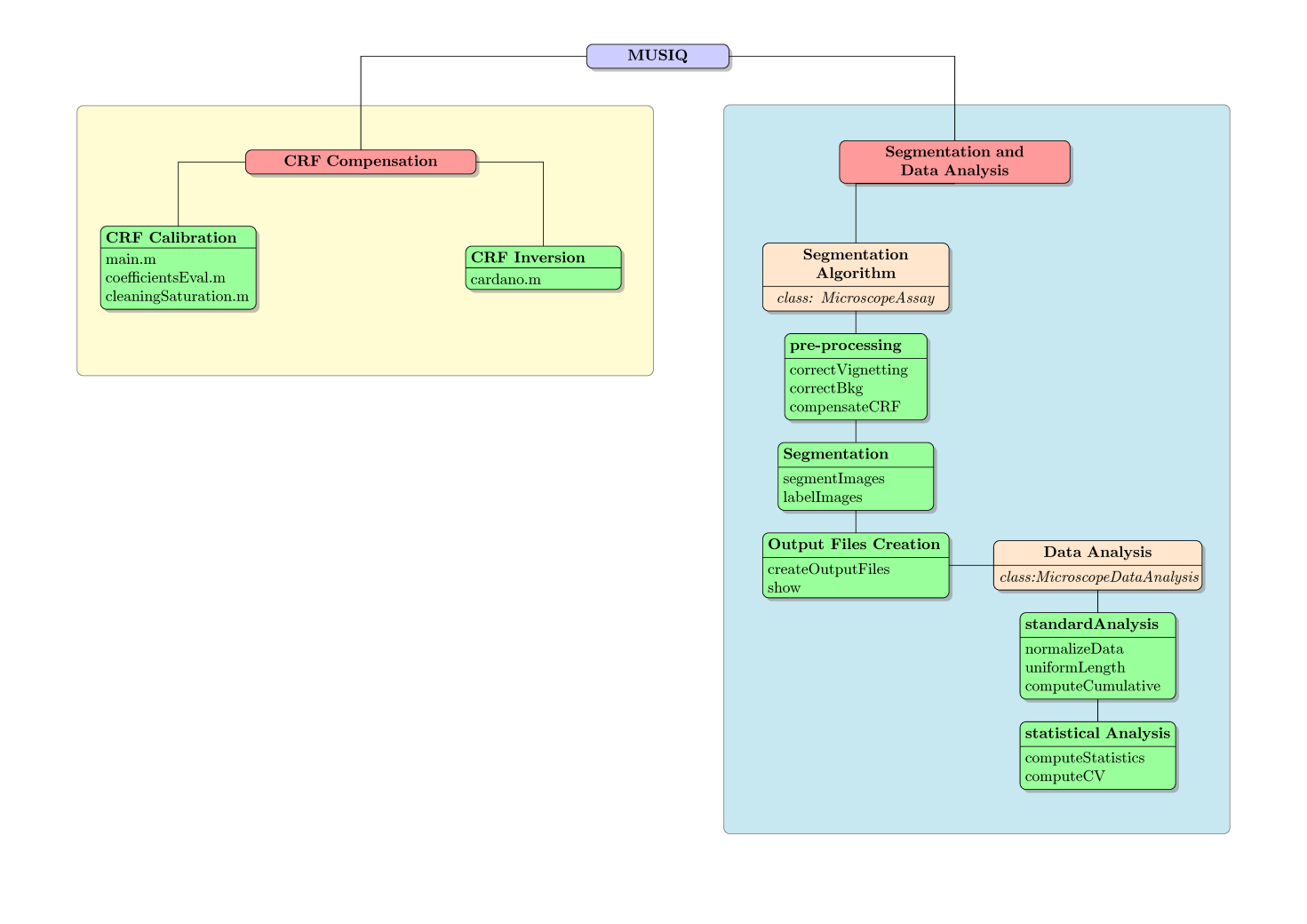


Matlab code for CRF compensation:

As detailed in the main text, the method used to compensate for the CRF is the radiometric self-calibration [1,2]. It involves acquiring multiple images of the same object at different exposure times and then fitting the intensity variation with a polynomial function. Since the radiance of the scene does not change, this procedure allows to identify the relation between the registered signal and the exposure time, thus determining the effect on the signal of the camera used to acquire it.

The section of the MUSIQ software that implements this aspect of the calibration procedure is coded in Matlab and is composed of a script and some custom made functions to use in combination with the regu toolbox [3]. The whole analysis can be executed by running the script ('main.m') with the appropriate parameters. The user needs to instantiate the variable 'folderImages' with a string representing the path of the folder containing the images, the variable 'exps' with a vector containing the exposure times used to acquire the images and the variable 'tr' with the number of technical replicates acquired for each exposure time as detailed below in the code box.

clear

clc

close all

% set configuration parameters

addpath('SET_TO_ABSOLUTE_PATH_REGU_TOOLBOX')

folderImages= 'SET_TO_ABSOLUTE_PATH_OF_THE_FOLDER_CONTAINING_THE_IMAGES';

exps=['SET_TO_THE_ESPOSURE_TIMES_USED'];

exps_string=['SET_TO_THE_ESPOSURE_TIMES_USED_AS_STRINGS(for_graphical_purposes)'];

tr='SET_TO_THE_NUMBER_OF_TECHNICAL_REPLICATES';

main.m

The algorithm uses this information to load the images and average the technical replicates (if present). Successively the images are normalized, thus bringing all the intensities between 0 and 1.

At this point the function 'coefficientsEval.m' is executed, it contains the instructions that allow the identification of the coefficients of the CRF as described in [1,2]. Its arguments are 'images', the 3D matrix containing the images to analyze; 'exps', an array containing the exposure times used in the experiment, and 'N' that is the degree of the polynomial that the function uses to fit the CRF. Since this last parameter is not easily identifiable, the main script executes 'coefficientsEval.m' on all the integers between 1 and 10 (included) and then uses an error function to determine which value is the most suitable for N.

Before estimating the coefficients of the CRF, 'coefficientsEval.m' executes the function 'cleaningSaturation.m' that analyzes the set of images and excludes those pixels that present undesired characteristics, for the current analysis.

Specifically all the pixels that reach saturation or present a non-monotonic relation with the exposure time might lead to an erroneous reconstruction of the CRF and thus are excluded from the subsequent analysis. When acquiring images for the CRF calibration, thus care must be taken not to use exposure times that cause a significant number of pixels to saturate as it will significantly reduce the data effectively employed in the analysis.

The coefficients of the CRF are then computed, as described in [1,2], the only adjustment made during this analysis is the application of the Tikhonov regularization method [3] to reduce the condition number of the coefficient's matrix.

The parameters of each polynomial function identified with this analysis are saved in a matrix that is successively analyzed to determine the function that best approximates the experimental data. This is done by comparing the ratio between two consecutive exposure times to the one estimated from the images and determining which polynomial is associated to the smallest error. In our case, the CRF was approximated with a third degree function that has a total error of 0.066 over the tested exposure times.

To invert this polynomial, and determine the radiance values corresponding to each gray-level of the image, we applied the Cardano's method. Specifically we created the Matlab function 'cardano.m', that uses a vector containing the coefficients of a third degree polynomial, to compute a matrix containing, in each column, one of the possible solutions of the provided equation (see example below).

solution=cardano([a,b,c]); being y=ax2+bx+c

Given the characteristics of the problem, the only solution physically sound is the one that is always positive, thus 'cardano.m' creates a text file, named CRF.txt, containing the values of the function associated to this solution evaluated at the 256 possible intensities of the 8-bit images. 'cardano.m' is applicable only to cubic equations, however other mathematical formulations exist for quadric and quartic equations.

Segmentation Code:

The class 'MicroscopeAssay' of the MUSIQ software collects a set of functions that can be used to segment the images acquired with an optical microscope and quantify the fluorescent signal emitted by each cell.

The constructor of this class expects as input a string representing the absolute path of the folder containing the images to analyze, as exemplified in the code box below). The remaining three arguments: 'replicates', 'imageDims' and 'volumePerSlide' need to be provided only to overload the default values: 2, 1024 x 1280 and 3 µL respectively.

M=musiq.MicroscopeAssay('./path/_c,tP_iC_bioR_expT_cF',replicates=2,

imageDims=[1024,1280],volumePerSlide=3)

In order for the system to load and properly elaborate the images, they need to be stored in 8-bit tiff format and coded in the RGB color space. Furthermore, they need to be organized in a folder structure that separates them according to biological replicate, circuit, level of induction and acquisition time point. In particular, each folder name needs to be encoded by following the scheme [ *_d_c,tP_iC_bioR_expT_cF* ], where:

*- d* is the experiment date,

*- c* is the identification string of the gene circuit and

*- tP* identifies the acquisition time point.

*- iC* represents the inducer concentration,

*- bioR* identifies the biological replicate,

*- expT* indicates the shutter speed,

*- cF* is the factor by which the volume of the culture was reduced before acquisition.

This allows for the automatic identification of the main characteristics of the experiment, the correct association between data and experimental condition, the determination of culture density and the application of the appropriate correction for different exposure times.

Launching the constructor loads the images in a data structure recognized by the other functions of the library.

The analysis then proceeds, as described in the main text, with the pre-elaboration of the images. This can be performed either through the pre-elaboration function ('MicroscopeAssay.preProcess'), whose invocation is reported below or by calling individually each correction function in the appropriate order.

M.preProcess()

'MicroscopeAssay.preProcess' is a wrapper function that has been created to facilitate and standardize the pre-elaboration phase and it calls the following functions in the presented order:

1. 'MicroscopeAssay.correctVignetting': function responsible for the vignetting correction, implemented as previously detailed. It requires the presence of a text file, named 'Vignetting.txt' in the same path as the code. This file contains a matrix, of the same size of the images in input, representing the vignetting image.
2. 'MicroscopeAssay.correctBkg', that applies the correction for the background as described in [4]. It is called iteratively, changing the vignetting corrected image given as argument.
3. 'MicroscopeAssay.compensateCRF' is the function that substitutes to the gray levels of the image the corresponding radiance levels, as determined by inverting the CRF identified during the calibration step. It has as input the image to correct, and thus it is executed iteratively on every background-corrected image. As previously mentioned the 256 possible radiance levels are saved in a text file, named 'CRF.txt' and saved in the folder containing the code, that this function loads and uses as a look-up table.

The successive step in the analysis consists in segmenting the images and quantifying the intensity of the signal emitted by each cell. The function 'MicroscopeAssay.segmentImages' accomplishes both these functions and, by leaving the argument 'output' to the default value (True), the execution of this function leads to the production of the output files (see example below).

M.segmentImages(output=True)

As previously mentioned in the main text, the segmentation algorithm initially applies a gaussian smoothing, to reduce the detection of the spurious edges, and then executes the zero-crossing edge detection algorithm. As a result the edges of the bacterial cells are identified. A hole-filling procedure and a BW- erosion complete the segmentation.

At this point 'MicroscopeAssay.segmentImages' calls the function 'MicroscopeAssay.labelImages' that labels the segmented image, assigning to each cell a progressive number, and computes the fluorescent signal at single cell level, by averaging the intensities of the pixels belonging to the same cell and applying the appropriate correction for the exposure time.

Finally if the argument 'output' is True, the function 'MicroscopeAssay.createOutputFiles' is executed. It creates two text files, one containing the fluorescence intensities emitted by each cell, organized by date, gene circuit, induction level (i.e. experimental condition) and time point, and the other reporting the cellular densities, computed as the number of segmented cells divided by the volume of culture used to prepare the slide.

The third output of the MUSIQ software, a pdf file containing the images at different stages of elaboration, is produced by the execution, after 'MicroscopeAssay.segmentImages' of the function 'MicroscopeAssay.show' that requires as input the stream that can be used to save the images, as shown in the code box below.

pdf_stream=matplotlib.backends.backend_pdf.PdfPages('document.pdf')

M.show(self, pdf_stream)

The cellular densities estimated with MUSIQ show a very good agreement with the corresponding OD_600_ values (R^2^=0.996, Fig. S2).


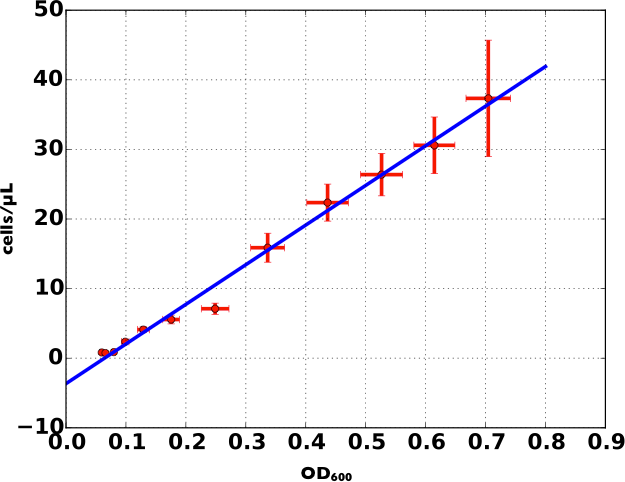


This result was obtained observing the growth of two bacterial population, that constitutively produce GFP and differ only for the promoter's strength, over a period of 4 hours with both the microscopy set-up and a plate reader (Tecan Infinite M200).

The OD_600_, the most widespread measure of cell density, was compared to the average number of segmented cells divided by the volume of culture used to prepare the slide. For each time point were acquired an average of 16 images while the OD_600_ measurements are in triplicates. The linearity of the relation between cells/µL and optical density, demonstrates how MUSIQ is able, not only to reliably quantify the fluorescent signal emitted by single bacterial cells on a microscope slide, but also to correctly estimate the culture's density.

Fluorescence Data Analysis:

The analysis of the fluorescence data is realized by the class 'MicroscopeDataAnalysis' of the MUSIQ software. The constructor of the class loads the text files created by 'MicroscopeAssay.segmentImages' through 'MicroscopeAssay.createOutputFiles', and organizes the data in the format recognized by the other functions of the library. The arguments required to create an object of the 'MicroscopeDataAnalysis' class are i) a string representing the absolute path of the folder containing the text files ('folder') and ii) a list containing the dates of the experiments to consider ('dates'), represented as strings (see code box below).

M = musiq.MicroscopDataAnalysis('./path/',['date1', 'date2…, dateN])

Unlike image segmentation, where the procedure is standard, the analysis of the data is more customizable and adaptable to the specific experiment. The only required step is the normalization of the data, executed running the function 'MicroscopeDataAnalysis.normalizeData'. This function has as input 'construct' and 'condition' that are strings identifying, respectively, the normalizer circuit and the experimental condition (induction level) used as reference and returns a dictionary containing the normalized data (as exemplified below). If only one of the acquired time points needs to be considered for the calibration, it can be specified through the parameter 'time', whose default value is 'all'.

normalizedData=M.normalizeData('SET_TO_ID_NORMALIZER',

'SET_TO_NORMALIZER_CONDITION', time='all')

Another function that is advisable to run, before comparing the fluorescence intensities of different experimental conditions, is 'MicroscopeDataAnalysis.uniformLength' that uniforms the cardinality of the populations available for different experimental conditions. Its arguments are 'data', a dictionary containing the fluorescence data and 'length' that specifies the cardinality of the populations in the output structure. The default value for this parameter is 'max' that corresponds to considering the most numerous populations possible, given the specified dataset, but any number below this value can be used for this parameter. An example of how to invoke this function is reported in the following code box.

equalizedData=M.uniformLength(normalizedData,length='max')

A very useful function coded in this library is 'MicroscopeDataAnalysis.computeCumulative' that combines the biological replicates by concatenating data of the same gene circuit, acquired in the same experimental condition and at the same time point but in different days (see example below).

cumulativeData=M.computeCumulative(equalizedData)

Since the three functions previously described ('MicroscopeDataAnalysis.normalizeData', MicroscopeDataAnalysis.uniformLength', 'MicroscopeDataAnalysis.computeCumulative') are generally part of the data analysis workflow, the MUSIQ software contains a wrapper function, named 'MicroscopeDataAnalysis.standardAnalysis' (whose invocation is reported below) that executes them sequentially and returns only the output of the last function ('MicroscopeDataAnalysis.computeCumulative') that is a dictionary containing the experimental data organized by genetic construct, experimental condition and time point. The inputs of this function are construct, condition and length that are the arguments of MicroscopeDataAnalysis.normalizeData' and 'MicroscopeDataAnalysis.uniformLength', respectively.

cumulativeData=M.standardAnalysis(SET_TO_ID_NORMALIZER',

'SET_TO_NORMALIZER_CONDITION','SET_TO_DATA_FINAL_CARDINALITY')

However the main function of this class is 'MicroscopeDataAnalysis.computeStatistics' that calculates average value, standard deviation, skewness and kurtosis of the dataset in input. If the other argument of the function, 'orderCriteria', is left to the default value ('construct') the structure of the output dictionary is the same as the input data, while by setting it to 'statisticalVariables' the keys of the output dictionary are: 'inducer', 'ave', 'std', 'count', 'skewness' and 'kurtosis' and the corresponding values are lists containing the results of the computation (see a more detailed example below). This second formulation is more convenient for the graphical representation of the results, however it doesn't allow for multiple gene circuits to be analyzed at the same time. In this case this function requires other two arguments: 'construct' and 'time' that represent, respectively, the string identifying the gene circuit and another string representing the time-point of interest.

meanDict,stdDict,ncellDict,skewDict,kurtDict=computeStatistics(

cumulativeData,orderCriteria='construct')

statsDict=computeStatistics(cumulativeData,orderCriteria='statisticalVariables',

circuit='SET_TO_CIRCUIT_ID',time='SET_TO_TIMEPOINT')

The function 'MicroscopeDataAnalysis.computeCV' has the same specifics of 'MicroscopeDataAnalysis.computeStatistics' but its outputs are two dictionaries containing the coefficient of variation (CV) and the coefficient of variation squared (CV^2^) of the dataset, or of the selected gene circuit, if 'orderCriteria' is set to 'statisticalVariables', as exemplified in the code box below.

The functions presented in this appendix are anticipated to be useful for a potential user. However a complete description of both the classes is reported in the corresponding reference manuals, available @www.mcebeng.it\ MUSIQ.

CVDict, CV2Dict =computeCV(

cumulativeData,orderCriteria='construct')

CVDict,CV2Dict=computeCV(cumulativeData,orderCriteria=

'statisticalVariables',circuit='SET_TO_CIRCUIT_ID',time='SET_TO_TIMEPOINT')

**References**

1 Mitsunaga T, Nayar SK Radiometric self calibration. In: Computer Vision and Pattern Recognition, IEEE Computer Society Conference on. Fort Collins, CO; 1999. p. 380-384.

2 Bevilacqua A, Gherardi A, Carrozza L. A robust approach to reconstruct experimentally the camera response function. In: Masmoudi, DS, Djemal K, editors. Image Processing Theory, Tools & Applications, IEEE Conference on; 2008. p. 340-345.

3 Hansen, PC. Matlab toolbox for analysis and solution of discrete ill-posed problems.1998. <http://www.mathworks.com/matlabcentral/regtools>. Accessed 3 Oct 2014.

4 Singh P, Garg AK. Morphology based non uniform background removal for particle analysis: A comparative study. International Journal of Computing and Corporate Research.  2011.

**Figure legends**

Fig S1: Schematic representation of the MUSIQ software library. The block on the right describes the workflow that must be followed to segment the images and analyse the data, while the one on the left reports the tools included in MUSIQ for the identification of the CRF.

Fig S2. Correlation plot showing the good agreement between the cellular density measured with MUSIQ (an average of 16 images/time point) and the optical density evaluated with the plate reader (in triplicates).
